# A novel N6-methyladenosine (m6A)-dependent fate decision for the lncRNA *THOR*

**DOI:** 10.1038/s41419-020-02833-y

**Published:** 2020-08-13

**Authors:** Hongmei Liu, Yuxin Xu, Bing Yao, Tingting Sui, Liangxue Lai, Zhanjun Li

**Affiliations:** 1 Key Laboratory of Zoonosis Research, Ministry of Education, Jilin University, 130062 Changchun, China; 2 CAS Key Laboratory of Regenerative Biology, Guangdong Provincial Key Laboratory of Stem Cell and Regenerative Medicine, South China Institute for Stem Cell Biology and Regenerative Medicine, Guangzhou Institutes of Biomedicine and Health, Chinese Academy of Sciences, 510530 Guangzhou, China; 3 Guangzhou Regenerative Medicine and Health Guang Dong Laboratory (GRMH-GDL), 510005 Guangzhou, China; 4 Institute for Stem Cell and Regeneration, Chinese Academy of Sciences, 100101 Beijing, China

**Keywords:** RNA, Cancer, Non-small-cell lung cancer

## Abstract

Previous studies have revealed the critical roles of the N6-methyladenosine (m6A) modification of long non-coding RNAs (lncRNAs) in cancers, but the relationship between the oncogenic role of the lncRNA *THOR* (a representative of cancer/testis lncRNAs) and m6A modification remains unclear. Here, we show that the internal m6A modification of the lncRNA *THOR* via an m6A-reader-dependent modality regulates the proliferation of cancer cells. Our findings demonstrated that the loss of the lncRNA *THOR* inhibits the proliferation, migration, and invasion of cancer cells in vitro and in vivo. In addition, m6A is highly enriched on lncRNA *THOR* transcripts, which contain GA (m6A) CA, GG (m6A) CU, and UG (m6A) CU sequence motifs. RIP-qRT-PCR and RNA pull-down assay results revealed that the specific m6A readers YTHDF1 and YTHDF2 can read the m6A motifs and regulate the stability of the lncRNA *THOR* (stabilization and decay). These m6A-dependent RNA-protein interactions can maintain the oncogenic role of the lncRNA *THOR*. Collectively, these findings highlight the critical role of the m6A modification in oncogenic lncRNA *THOR* and reveal a novel long non-coding RNA regulatory mechanism, providing a new way to explore RNA epigenetic regulatory patterns in the future.

## Introduction

Long non-coding RNA (lncRNA), which is an abundant and functionally diverse species of non-coding RNA (ncRNA), is more than 200 nt transcripts long with limited or no protein-coding capacity^1–5^. The categorization criteria of lncRNAs largely depend on their functional roles and conservation^6–8^, and the functions of lncRNAs in different biological stages is anectodal^9^. Recently, a novel lncRNA with expression limited to the testis and widespread expression in multiple cancer types was discovered and named cancer/testis lncRNA *THOR*^10^. Dysregulated expression of lncRNA *THOR* has been reported to modulate the progression of various types of cancers, such as melanoma, non-small cell lung cancer, osteosarcoma and renal cell carcinoma^10–12^. Currently, the molecular mechanisms of cancer/testis lncRNAs in cancer metastasis are largely unknown and need to be fully elucidated^10–13^.

Recently, the new field of “RNA epigenetics” has been booming^14,15^, and N6-methyladenosine (m6A) has been identified as a post-transcriptional regulatory mark in multiple RNA species, including messenger RNAs (mRNAs)^1,2,16^, transfer RNAs (tRNAs)^3,4,17–20^, ribosomal RNAs (rRNAs)^21^, small nuclear RNA^22^, small non-coding RNAs (sncRNAs)^23^, and lncRNAs^16,24^. It has been reported that the m6A RNA modification is conferred by methyltransferases (writers), such as methyltransferase-like 3 (METTL3), forming the catalytic core of the m6A methyltransferase complex^25–28^. In addition, the biological function of m6A is mediated through the recognition of the m6A site by m6A “readers”^1,29,30^, such as the YT521-B homology (YTH) family, including YTH domain family (YTHDF1-3) and the nuclear member YTH domain containing 1 (YTHDC1)^1,31–35^. These readers regulate RNA processing or metabolism, including mRNA alternative splicing, export, translation, and decay^1,29,30,36–38^. In addition, accumulated evidence has revealed that m6A modification plays important roles in circadian rhythms^39^, spermatogenesis^40^, embryogenesis, heat shock responses^41^, DNA damage response^42^, and cell pluripotency and reprogramming^43,44^. However, the relationship between the oncogenic role of the lncRNA *THOR* and m6A modification remains unclear.

Therefore, we are interested in determining whether the oncogenic role of the lncRNA *THOR* is associated with m6A modification or not and the accurate m6A modification sites in the lncRNA *THOR*. In this work, we elucidated the oncogenic role of m6A modification of the lncRNA *THOR* in promoting cancer cell proliferation. In addition, we found that the m6A readers YTHDF1 and YTHDF2 may play a role in balancing the gene transcription and decay of the lncRNA *THOR*.

## Results

### Significantly reduced cell proliferation in the h*THOR*^−/−^ cells

Previous studies indicated that the lncRNA *THOR*, representative of cancer/testis lncRNAs, plays a positive role in cancer cell proliferation^10–13^. To explore the function of the lncRNA *THOR* in cancer cells, the cellular distribution of the lncRNA *THOR* was analysed by qRT-PCR and RNA-FISH assay. The results showed that lncRNA *THOR* transcripts were abundant in the cytoplasm of H1299 cells (Fig. S1A–C). In addition, dramatic reductions in cell proliferation, colony formation and colony size in soft agar were observed in the lncRNA *THOR*-knockdown H1299 cells (si*THOR*) (Fig. S1D, E) compared to levels in the siNC controls. The Transwell migration and invasion assays demonstrated the significantly decreased migration and invasion of the si*THOR* cells compared with the siNC control cells (Fig. S1F).

**Fig. 1:**
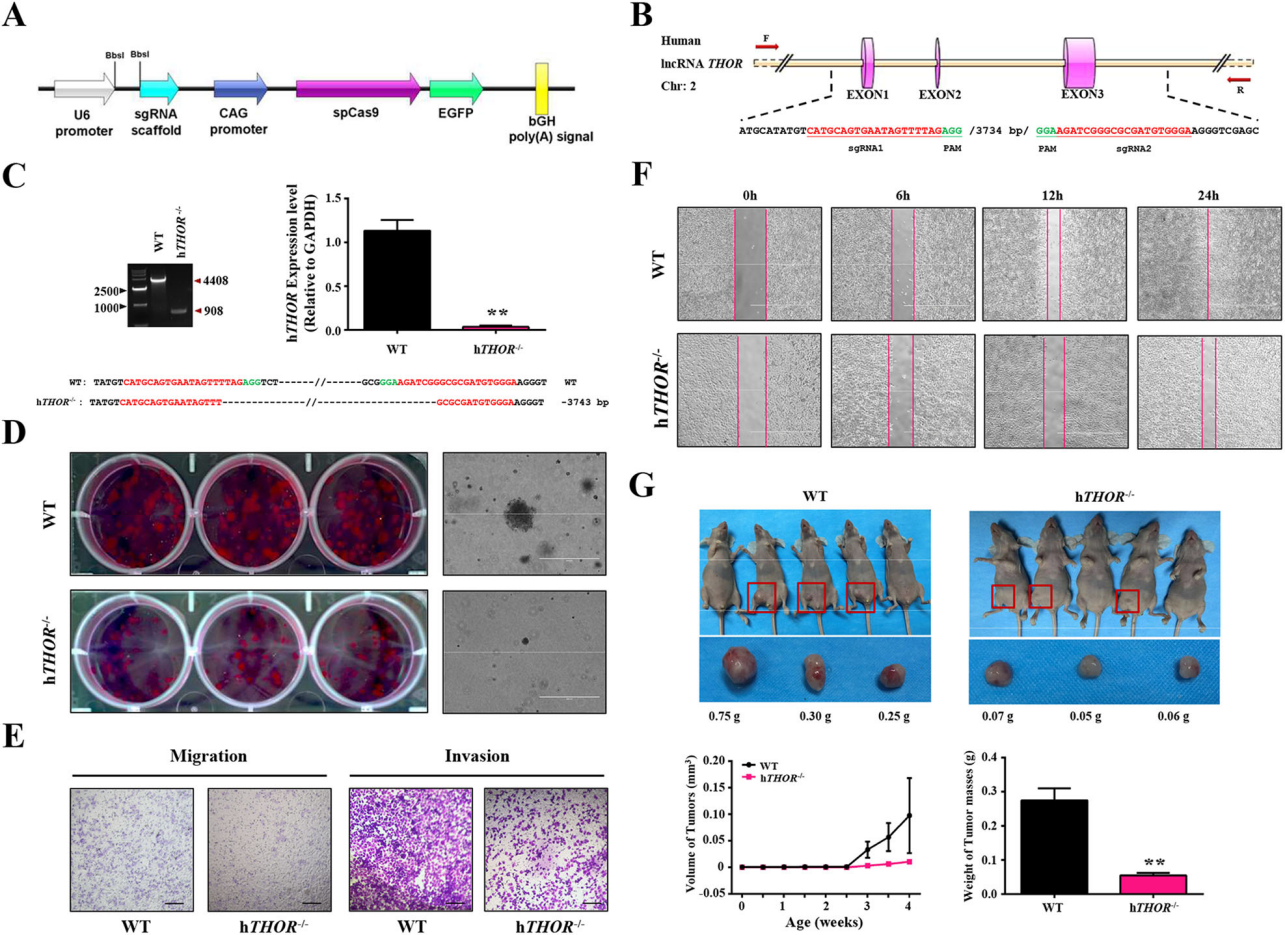
Significantly reduced cell proliferation in h*THOR*^−/−^ cells. **a** Schematic diagram of the PX458 plasmid used for lncRNA *THOR* gene knockout using the CRISPR/Cas9 technology. **b** Schematic diagram of sgRNA targeting the human lncRNA *THOR* gene loci. LncRNA *THOR* exons are indicated by cylinders, the target sites of the 2 sgRNA sequences (sgRNA1 and sgRNA2) are highlighted in red, and the protospacer-adjacent motif (PAM) sequence is highlighted in green. Primers F and R were used for detecting mutations in H1299 cells. **c**. The mutation detection of h*THOR*^−/−^ cells by PCR, Sanger sequencing, and qRT-PCR. LncRNA *THOR* expression was significantly decreased in KO cells compared with that of WT control cells (**p < 0.01). **d** Cell proliferation analysis of WT and h*THOR*^−/−^ cells by plate colony formation assays and soft agar assays. The results indicate that the growth of h*THOR*^−/−^ cells was decreased compared with WT cells. Scar bars, 400 μm. **e** Representative image of the migration assays and invasion assays, implying that the migration and invasion abilities of h*THOR*^−/−^ cells were reduced compared with WT cells. Scar bars, 50 μm. **f** Representative images of the wound-healing assay in h*THOR*^−/−^ cells and control cells. The scratch was measured 6, 12 and 24 h after it was initially made. The results showed downregulation of the migration healing ability of h*THOR*^−/−^ cells compared with their WT control cells. Scar bars, 1000 μm. **g** Tumour xenograft assays were used to detect the tumourigenesis of h*THOR*^−/−^ cells and control cells. (Top left and right) Photos of subcutaneous tumours in the h*THOR*^−/−^ group and in the WT control group. (Bottom left) The subcutaneous tumour growth in the h*THOR*^−/−^ group was slower than it was in the WT control group, which was determined based on the tumour (calculated based on weekly measurements after injection). (Bottom right) In this panel, the weight of tumour mass was measured and compared with the control groups. The results showed that H1299 cell growth was inhibited by lncRNA *THOR* knockout in vivo (**p < 0.01).

Furthermore, a *THOR*-knockout (h*THOR*^−/−^) H1299 cell line was generated via CRISPR/Cas9 technology and paired single-guide RNAs (sgRNAs) (Fig. 1a, b). The complete gene knockout (KO) of the lncRNA *THOR* in the H1299 cells was confirmed by DNA sequencing and qRT-PCR (Fig. 1c). Functional experiments showed that the h*THOR*^−/−^ cells showed decreased colony, migration, invasion, and wound-healing abilities (Fig. 1d–f). Additionally, the significantly reduced proliferation of the h*THOR*^−/−^ cells was discovered, compared to that of the wild-type (WT) control cells, in the murine tumour xenograft models (Fig. 1g). Furthermore, the decreased expression of oncogenes (*Myc*, *Igf2*, *Gli1*, *Kras* and *Cd44*) was determined in the h*THOR*^−/−^ cells (Fig. S1G). These results demonstrated that the lncRNA *THOR* has an oncogenic role in the proliferation of H1299 cells, a finding consistent with that of a previous study^10^.

### m6A modifications were enriched in the lncRNA *THOR*

**Fig. 2:**
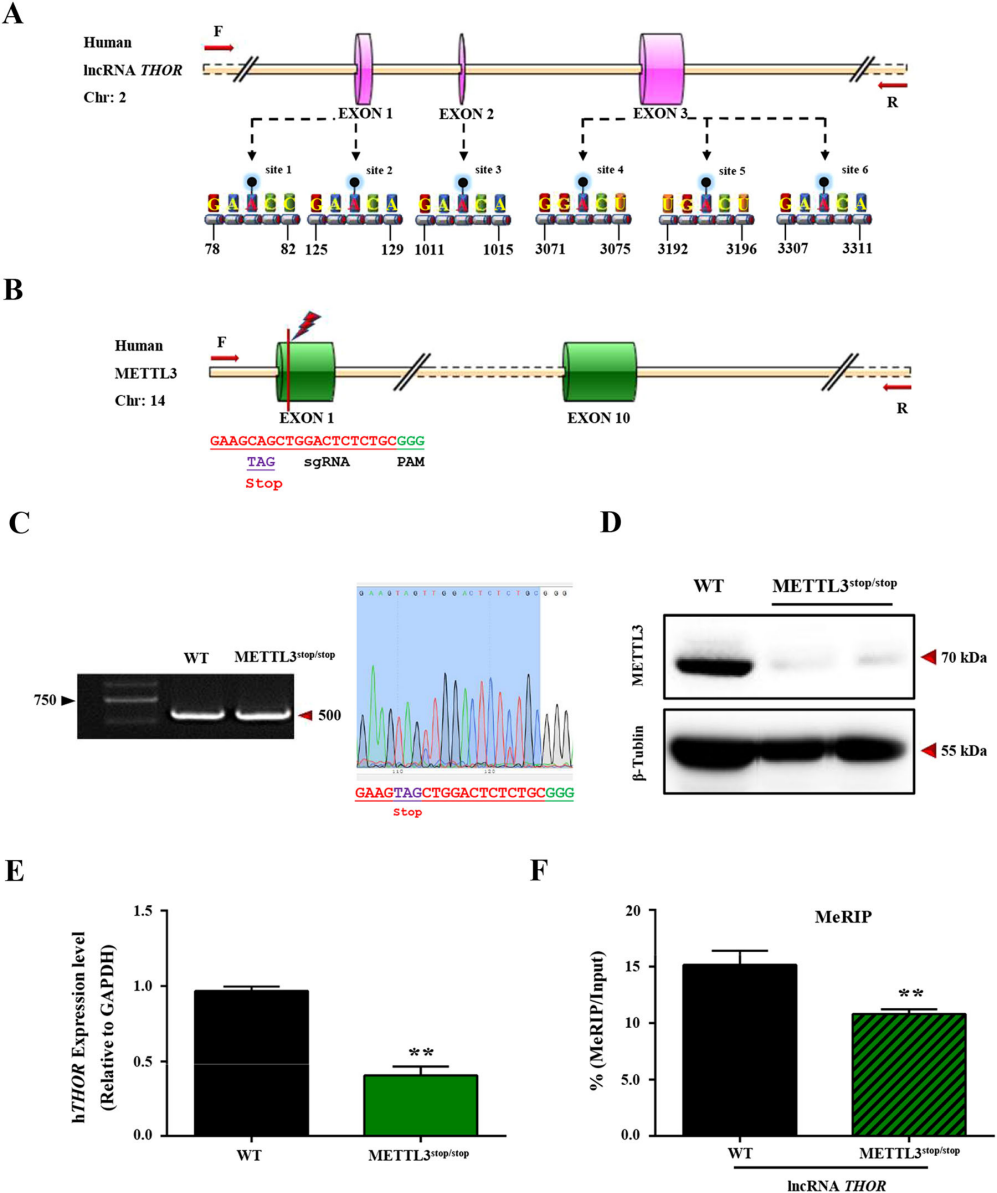
m6A modification were enriched in the lncRNA *THOR*. **a** The six m6A modification sites in lncRNA *THOR* were predicted by the online tool SRAMP (http://www.cuilab.cn/sramp/). **b** Knockout of METTL3 by BE4-Gam editing system. sgRNA (red), PAM region (green), target sites (red), and stop codon (underlined). **c** The genotype determination of METTL3^stop/stop^ cells by PCR conducts and Sanger sequencing. **d** Western blot results showed complete loss of METTL3 protein in METTL3^stop/stop^ cells. **e** Downregulated expression of lncRNA *THOR* in METTL3^stop/stop^ cells compared with that of control cells, which was determined by qRT-PCR assays (**p < 0.01). **f** Downregulated expression of lncRNA *THOR* in m6A-modified upon METTL3 depletion compared with that of control cells, which determined by MeRIP- qRT-PCR assays, the percentage of the input is shown (**p < 0.01).

Recent studies have suggested that m6A modifications can determine the fate of cancer cells, and thousands of cancer-specific transcripts, including lncRNAs, are modified by m6A^45^. To define the potential m6A modification sites in the lncRNA *THOR*, the gene sequence of the lncRNA *THOR* was aligned with the GEO database (GSE76367: MeRIP-seq analysis of METTL3-knockdown H1299 cells from a previous study^46^), and m6A motifs in lncRNA *THOR* were predicted by the online tool SRAMP (a sequence-based N6-methyladenosine (m6A) modification site predictor)^47^. The results showed that sites A80, 127, 1013, 3073, 3194 and 3309 were predicted to be m6A modification sites, and they were distributed in three exons of the lncRNA *THOR* (Fig. 2a).

METTL3 was originally identified as a methyltransferase critical for m6A modification^46^. To characterize the m6A methylation of lncRNA *THOR*, the stable METTL3-knockout H1299 cell line (METTL3^stop/stop^) was generated by the BE4-Gam-induced STOP codon (iSTOP) system (Fig. 2b–d). The significantly reduced gene expression of the lncRNA *THOR* (Fig. 2e) and the decreased m6A level on the lncRNA *THOR* in the METTL3^stop/stop^ cells were determined by qRT-PCR and methylated RNA immunoprecipitation (MeRIP) assay. The results indicated that the positive regulatory role of the lncRNA *THOR* is mediated in an m6A-dependent manner (Fig. 2f).

### The m6A modification mediated the proliferation of the cells expressing the lncRNA *THOR*

**Fig. 3:**
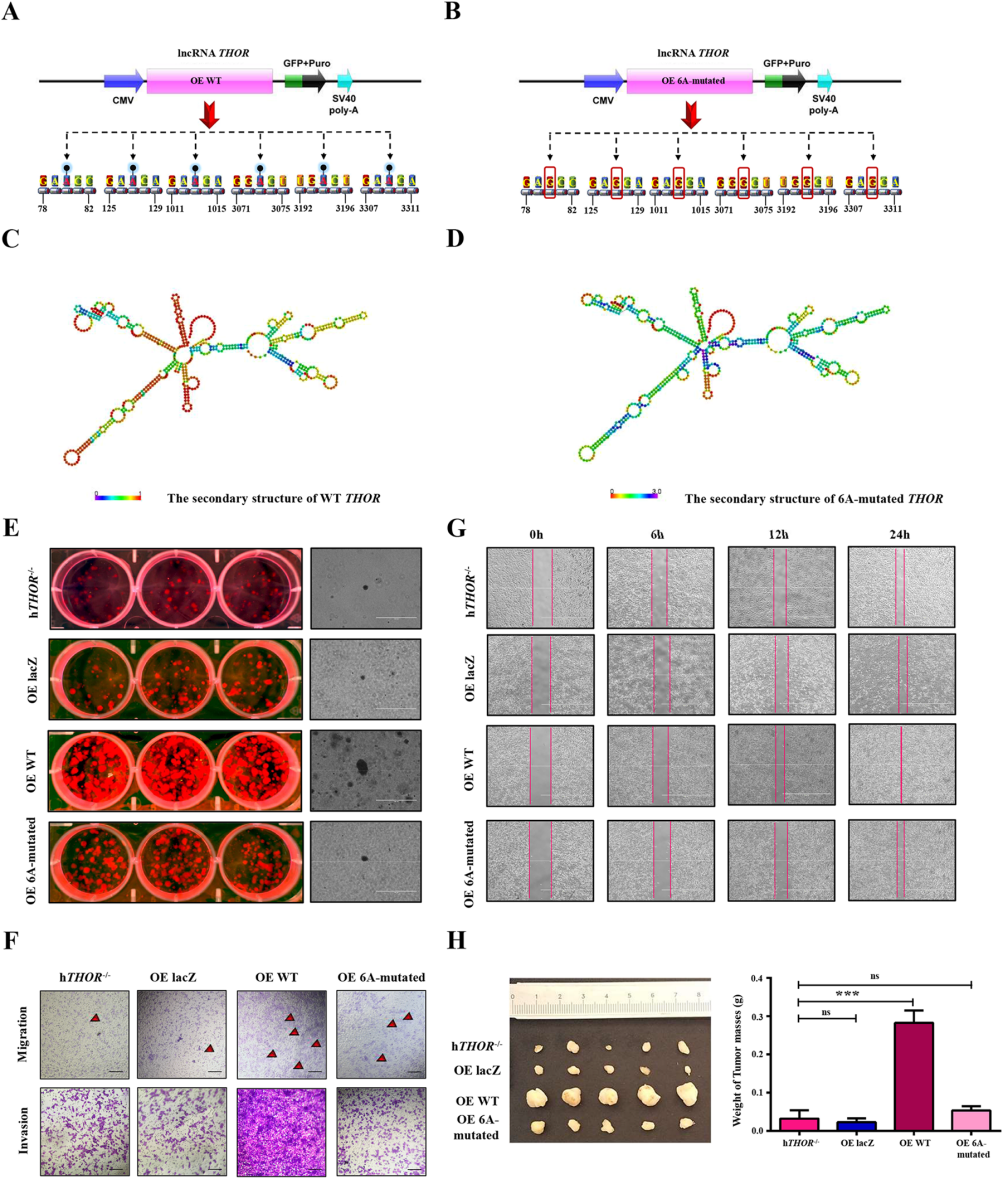
The m6A modification mediated the proliferation of the cells expressing the lncRNA *THOR*. **a** Schematic diagram of the overexpression WT lncRNA *THOR* plasmid. **b** Schematic diagram of the OE 6A-mutated plasmid. **c** The secondary structure of WT lncRNA *THOR* was predicted (http://rna.tbi.univie.ac.at/). The red color indicates strong confidence for the prediction of each base. **d** The secondary structure of OE 6A-mutated lncRNA *THOR* was predicted (http://rna.tbi.univie.ac.at/). The red color indicates strong confidence for the prediction of each base. **e** Cell proliferation analysis of h*THOR*^−/−^, OE lacZ, OE WT and OE 6A-mutated cells by plate colony formation assays and soft agar assays. The results indicate that OE 6A-mutated cells grow more slowly than OE WT cells. Scar bars, 400 μm. **f** The migration and invasion of h*THOR*^−/−^ cells, OE lacZ cells, OE WT cells and OE 6A-mutated cells were analysed using polycarbonate membrane inserts in a 24-well plate. The results indicate that the migration and invasion abilities of OE 6A-mutated cells were reduced compared to those of OE WT cells. Scar bars, 50 μm. **g** Wound healing assay in h*THOR*^−/−^, OE lacZ, OE WT and OE 6A-mutated cells. The scratch was measured 6, 12, and 24 h after it was initially made. The results showed that downregulation of migration healing ability in OE 6A-mutated cells compared with that of OE WT cells. Scar bars, 1000 μm. **h** Tumour xenograft assays were used to detect the tumourigenesis of h*THOR*^−/−^, OE lacZ, OE WT and OE 6A-mutated cells. The subcutaneous tumour size in the OE 6A-mutated group was smaller than it was in the OE WT group, and the average weight of tumour mass weight was less in the OE 6A-mutated group than it was in the OE WT group. Implying that the in vivo growth ability of OE 6A-mutated cells was weaker than that of OE WT cells (^***^p < 0.001, ns denotes not significant).

To further assess the relationship between the six predicted m6A sites and the regulatory functions of the lncRNA *THOR*. Three overexpression (OE) vectors were constructed, including wild-type (WT) lncRNA *THOR* (OE WT), mutated lncRNA *THOR* (the adenine residues of the six predicted m6A sites A80, 127, 1013, 3073, 3194 and 3309-G were replaced by guanine residues, OE 6A-mutated), and negative control (OE lacZ). Then, the stably overexpressed cell lines were generated using the PiggyBac transposon system (Fig. 3a, b and S2A). The qRT-PCR results showed that the significantly reduced expression of the lncRNA *THOR* in the OE 6A-mutated cells was compared with that in the OE WT cells (Fig. S2B). In addition, the expression of *Myc*, *Kras*, *Gli1*, *Igf2*, and *Cd44* was remarkably decreased in the OE 6A-mutated cells compared with their expression in the OE WT cells (Fig. S2C).

Then, the results of the online prediction tool (http://rna.tbi.univie.ac.at/) showed that the secondary structure of the 6A-mutated lncRNA *THOR* was changed compared with that of the WT lncRNA *THOR* (Fig. 3c, d), which is consistent with the regulatory function of m6A being depending on RNA structure as shown in a previous study^30^. To further assess the relationship between the six predicted m6A sites and the regulatory functions of the lncRNA *THOR*, functional experiments were performed with the stable cell lines (h*THOR*^−/−^, OE lacZ, OE WT, and OE 6A-mutated cells). The results showed that the colony formation ability (Fig. 3e), migration and invasion ability (Fig. 3f), wound-healing ability (Fig. 3g) and subcutaneous tumour sizes (Figs. 3h and S2D) were significantly decreased in the OE 6A-mutated cells compared with these measures in the OE WT cells. Collectively, these results confirmed that the m6A modification enrichment of the lncRNA *THOR* is involved in regulating cancer cell proliferation in vitro and in vivo.

### The determination of accurate m6A modification sites on the lncRNA *THOR*

**Fig. 4:**
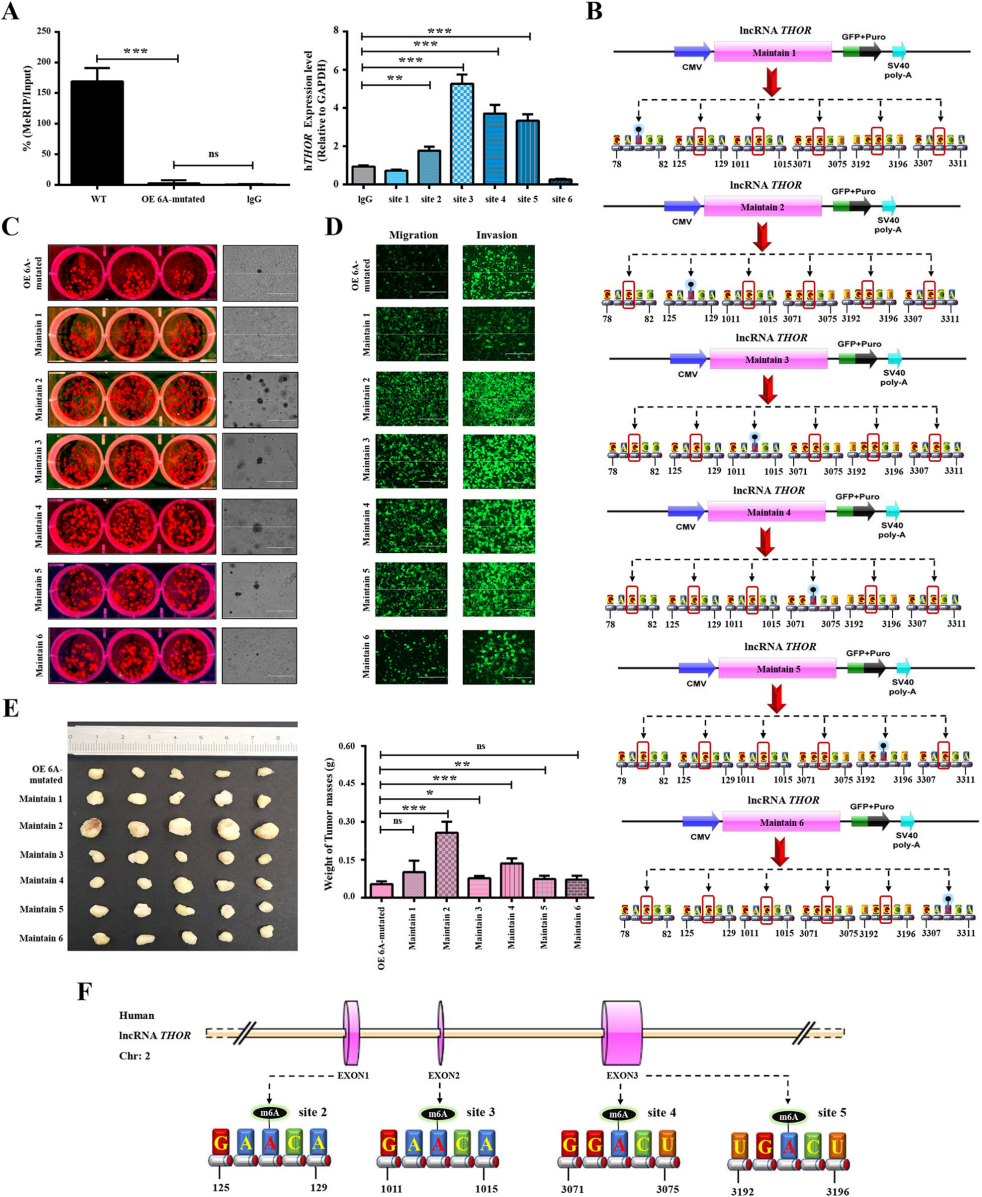
The determination of accurate m6A modification sites in lncRNA *THOR*. **a** m6A modification sites in lncRNA *THOR* were confirmed by MeRIP-qPCR. (Left) Purified RNA was analyzed by qRT-PCR. The results showed that the m6A protein-interacting mRNA of the WT group was obviously higher than that in the corresponding negative control group, but compared with the OE 6A-mutated group with the corresponding negative control group, there was no significant difference. (Right) qRT-PCR using specific primers (for each predicted site). The results show that in sites 2, 3, 4 and 5, the expression was higher than it was in the negative control. Thus, these results showed that four out six predicted sites (sites 2, 3, 4 and 5) were m6A modification sites. (**p < 0.01, ***p < 0.001; ns denotes not significant). **b** Schematic diagram of OE plasmids with different predicted m6A sites maintained (Maintain 1–6). **c** Plate colony formation assays and soft agar assays of OE 6A-mutated group and the Maintain 1–6 cell lines. The results indicate that Maintain 2, 3, 4 and 5 groups grew faster than the OE 6A-mutated group. Scar bars, 400 μm. **d** The migration and invasion of OE 6A-mutated group and the Maintain 1–6 cell lines were analysed using polycarbonate membrane inserts in a 24-well plate. The results indicate that the migration and invasion ability of Maintains 2, 3, 4 and 5 were increased compared with OE 6A-mutated. Scar bars, 400 μm. **e** Tumour xenograft assays were recruited to detect the tumourigenesis of the OE m6A mutated group and the Maintain 1–6 cell lines. **f** Schematic diagram of defined m6A modification sites on lncRNA *THOR* (sites 2, 3, 4 and 5).

First, a methylated RNA immunoprecipitation (MeRIP) assay and qRT-PCR were performed to verify the m6A modification sites on lncRNA *THOR*. The results showed that four of six predicted sites were m6A modification sites (including site 2, site 3, site 4 and site 5) of the lncRNA *THOR* (Fig. 4a). Then, plasmids with a single methylated adenine residue (which was predicted to be modified by m6A), named Maintain 1–6 (Fig. 4b), were transfected into h*THOR*^−/−^ cells using the PiggyBac transposon system, and the genotype was confirmed via PCR and Sanger Sequencing (Fig. S2A). The expression level of lncRNA *THOR* was calculated by qRT-PCR. As shown in Fig. S2E, the expression level of the lncRNA *THOR* was increased in the Maintain 1–6 cell lines (Maintain 4 > Maintain 5 > Maintain 1 > Maintain 3 > Maintain 2 > Maintain 6) compared with the level in the OE lacZ cell line.

Furthermore, significantly increased colony formation (Fig. 4c), migration and invasion ability (Fig. 4d), wound-healing ability (Fig. S3) and subcutaneous tumour sizes were also determined in the stably expressing Maintain 2–5 cell lines compared with the OE 6A-mutated cell line (Figs. 4E and S2F). These results revealed that four predicted m6A sites, located at 127, 1013, 3073 and 3194, in the lncRNA *THOR* (Fig. 4f) may play a role in cancer cell proliferation in an m6A-dependent manner.

### The functions of the m6A modification sites in the lncRNA *THOR*

**Fig. 5:**
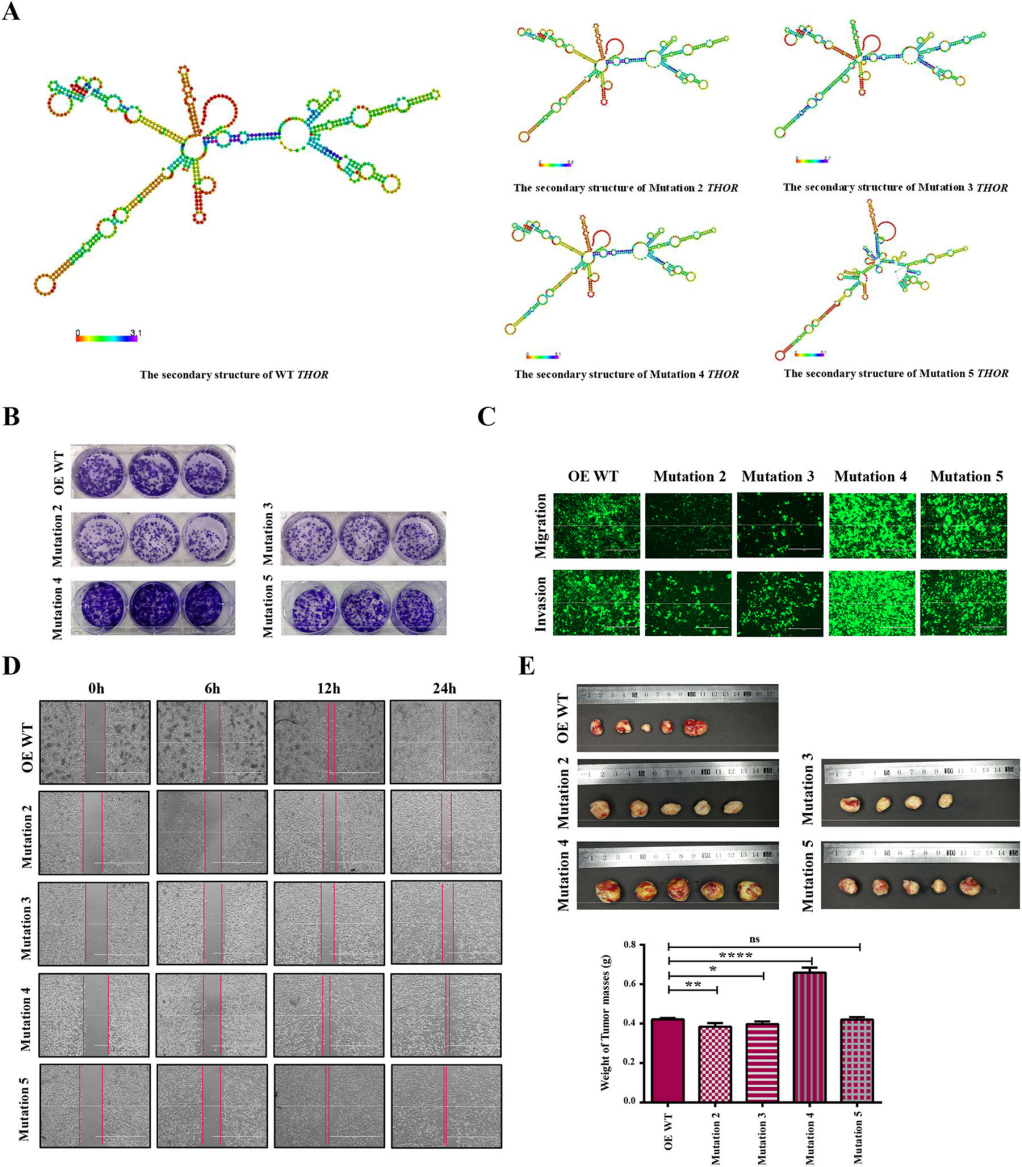
The function of m6A modification sites in lncRNA *THOR*. **a** The secondary structure of WT and Mutation 2–5 *THOR* were predicted (http://rna.tbi.univie.ac.at/). The red color indicates strong confidence for the prediction of each base. **b** Plate colony formation assays of OE WT cell line and the Mutation 2–5 cell lines. **c** The migration and invasion of OE WT cell line and the Mutation 2–5 cell lines were analysed using polycarbonate membrane inserts in a 24-well plate. Scar bars, 400 μm. **d** Wound healing assay in OE WT cell line and the Mutation 2–5 cell lines. The scratch was measured 6, 12 and 24 h after it was initially made. Scar bars,1000 μm. **e** Tumour xenograft assays were used to detect the tumourigenesis of mutant cell lines (Mutation 2, 3, 4 and 5). The subcutaneous tumour sizes in the Mutation 2 and 3 groups were smaller than they were in the OE WT group, the Mutations 4 tumours were larger than they were in the OE WT group, and the Mutations 5 tumours were similar with the OE WT group. Furthermore, the average weight of tumour mass weight in the mutant groups showed similar results. (*p < 0.05, **p < 0.01 and ****p < 0.0001; ns denotes not significant).

To assess the roles of different m6A modification sites in the lncRNA *THOR*, four overexpression vectors were constructed, in which the methylated adenine residue (A127, 1013, 3073 and 3194) was replaced by a guanine residue (A–G), named Mutation 2, Mutation 3, Mutation 4 and Mutation 5 (Fig. S4A). Then, the vectors were transfected into the h*THOR*^−/−^ cells using the PiggyBac transposon system. The genotype was confirmed via PCR amplification and Sanger Sequencing (Fig. S4B), and the gene expression of the lncRNA *THOR* was determined by qRT-PCR (Fig. S4C). The results showed that the expression level of the lncRNA *THOR* was increased in the Mutation 2–5 cell lines (Mutation 3 > Mutation 4 > Mutation 2 > Mutation 5) compared with that of the OE lacZ cell line. Furthermore, we predicted the secondary structure of Mutation 2, Mutation 3, Mutation 4 and Mutation 5 with the online tool (http://rna.tbi.univie.ac.at/), and the different secondary structures were determined in those mutant lncRNA *THOR* (Fig. 5a).

Interestingly, significantly inhibited colony formation (Fig. 5b), migration, invasion ability (Fig. 5c) and wound-healing ability (Fig. 5d) were detected in the stably expressing Mutation 2 and Mutation 3 cell lines, while significantly increased colony formation (Fig. 5b), migration, invasion (Fig. 5c) and wound-healing abilities were discovered in the stably expressing Mutation 4 and Mutation 5 cell lines (Fig. 5d). The analyses of the murine tumour xenografts confirmed these results in vivo (Figs. 5e and S4D).

Collectively, these findings indicated that modification at site 2 and site 3 of the lncRNA *THOR* promotes cancer cell proliferation, and modification at site 4 and site 5 of the lncRNA *THOR* inhibits cancer cell proliferation.

### The m6A readers YTHDF1 and YTHDF2 play balancing roles in regulating the gene transcription or decay of the lncRNA *THOR*

m6A is the first identified and most abundant internal modification of RNA that affects RNA metabolism and stability^48^. Here, the half-life of 6A-mutated lncRNA *THOR* was prolonged compared with that of the OE WT cell line (Fig. S5A), which is consistent with that m6A alters the stability (stabilization and degradation) of RNA in previous study^1^. Characterization of m6A readers has provided valuable insights into the molecular mechanisms of m6A-mediated post-transcriptional gene regulation^49^, including RNA processing, translation and decay. In addition, it has been demonstrated that YTHDF2 promotes RNA degradation in the cytoplasm in an m6A-dependent manner^50^, while YTHDF1 cooperates with the translation initiation machinery to enhance the translation efficiency of target RNAs in mammals^51–53^.

**Fig. 6:**
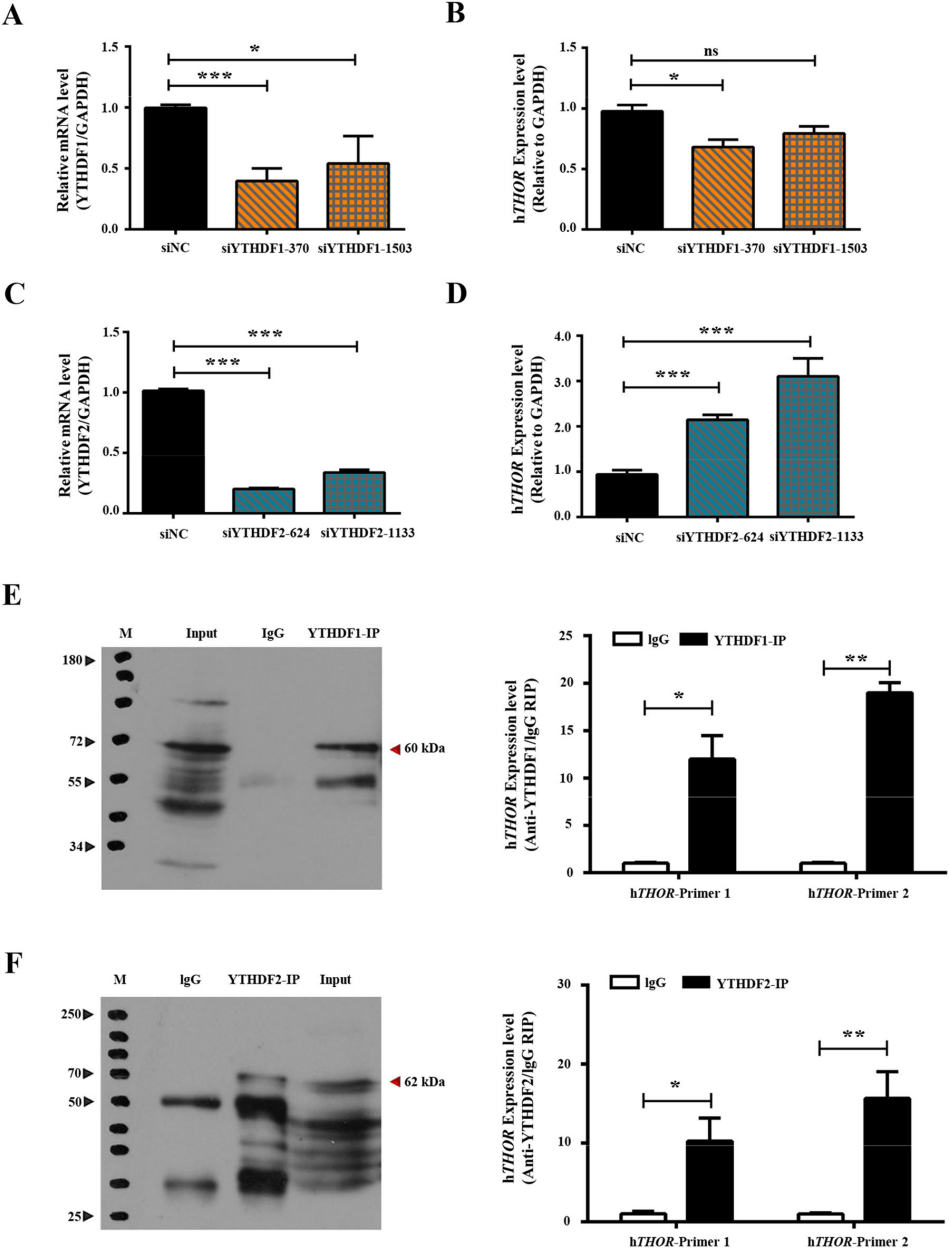
The m6A readers YTHDF1 and YTHDF2 play balancing role in regulating the gene transcription or decay of the lncRNA *THOR*. **a** The expression of YTHDF1 in siYTHDF1 H1299 cells and control cells was determined by qRT-PCR assays. The results showed that the expression of YTHDF1 was downregulated in siYTHDF1 cells compared with that of the control cells (*p < 0.05, ***p < 0.001). **b** The expression of lncRNA *THOR* in siYTHDF1 H1299 cells and control cells was determined by qRT-PCR assays. The results showed that the expression of *THOR* was downregulated in siYTHDF1 cells compared with that of the control cells (*p < 0.05, ns denotes not significant). **c** The expression of YTHDF2 in siYTHDF2 H1299 cells and control cells was determined by qRT-PCR. The results showed that the expression of YTHDF2 was upregulated in siYTHDF2 cells compared with that of control cells (***p < 0.001). **d** The expression of lncRNA *THOR* in siYTHDF2 H1299 cells and control cells was determined by qRT-PCR. The results showed that the expression of lncRNA *THOR* was upregulated in siYTHDF2 cells compared with that of control cells (***p < 0.001). **e** The interaction between lncRNA *THOR* and YTHDF1 was determined by RIP-qPCR assay. Western blotting assays showed that the antibody was specific, and the interaction between lncRNA *THOR* and YTHDF1 was confirmed by qRT-PCR (*p < 0.05, **p < 0.01). **f** The interaction between lncRNA *THOR* and YTHDF2 was determined by RIP-qPCR assay. Western blotting assays showed that the antibody was specific, and the interaction between lncRNA *THOR* and YTHDF2 was confirmed by qRT-PCR (*p < 0.05, **p < 0.01).

First, the qRT-PCR results confirmed the significantly decreased expression level of the lncRNA *THOR* in the YTHDF1-knockdown cell line (Fig. 6a, b) but increased expression level in the YTHDF2-knockdown cell line compared with the siNC control cell line (Fig. 6c, d). Next, to investigate the binding of YTHDF1 and YTHDF2 to the lncRNA *THOR* transcripts, the transcript-specific binding RNA-protein complexes were immunoprecipitated with YTHDF1 or YTHDF2 antibodies and then analysed via Western blotting and qRT-PCR. The results indicated that the lncRNA *THOR* was enriched in the YTHDF1-IP (Fig. 6e) and YTHDF2-IP (Fig. 6f) groups but not in the IgG group (negative control). Additionally, the RNA decay assays demonstrated that the m6A modification at sites 4 and 5 promotes the degradation of the lncRNA *THOR* (Fig. S5B). Collectively, YTHDF1 and YTHDF2 can read the m6A modification on the lncRNA *THOR*.

The expression of lncRNA *THOR* was decreased in the Maintain 2 and Maintain 3 cell lines after YTHDF1 interference compared with that of the siNC cell line (Fig. S5C, D). In addition, the significantly increased stability of the lncRNA *THOR* was detected in the Maintain 4 and Maintain 5 cell lines after YTHDF2 interference, compared with that of the siNC control cell line (Fig. S5E, F).

These results suggest that YTHDF1 and YTHDF2 cooperate by binding different motifs to regulate the function of *THOR* via a “*THOR*-m6A-reader” mechanism, and these readers may play a balancing role in regulating the gene transcription or decay of the lncRNA *THOR*.

## Discussion

The oncogenic role of lncRNA *THOR* has been determined in liver cancer^54,55^, renal carcinoma cells^12^, osteosarcoma^11^, nasopharyngeal carcinoma cells^56^, tongue squamous cells^57^, and colon cancer cells^58^ in previous studies. In this study, the h*THOR*^−/−^ H1299 cell line was generated via CRISPR/Cas9 technology using paired single-guide RNAs (sgRNAs) instead of RNA interference, which is not a complete gene knockout system. Our results suggested that h*THOR*^−/−^ could be used to inhibit cancer cell colony formation, migration, invasion-promoting, wound-healing capacities and the formation of murine tumour xenografts. In addition, the function of lncRNA *THOR* correlated with m6A methylation was also investigated for the first time, highlighting that YTHDF1 and YTHDF2 recognize the different m6A modification sites of lncRNA *THOR*. To the best of our knowledge, this is the first report determining the function of m6A methylation in lncRNA *THOR* and the balancing role of YTHDF1 and YTHDF2 in regulating the stabilization and degradation of lncRNA *THOR*.

Previous studies have shown that lncRNAs are regulated by the recruitment of miRNAs^15^ and that stability is enhanced by the accumulation of m6A modifications^59^, with the binding of low-complexity proteins, interactions with m6A readers, and RNA translation, splicing, stability, and decay additional regulatory mechanisms^1,29,36–38,60^. It has been shown m6A modification of linc1281 affected the binding of let-7 to linc1281 in embryonic stem cells (ESCs)^15^. Another report indicated that the m6A modification of lncRNA RP11 can promote the propagation of colorectal cancer (CRC) cells via post-translational upregulation of Zeb1^61^. However, no study has focused on the functions of m6A of the lncRNA *THOR*. In this study, m6A enrichment was evidenced by the sequence of the lncRNA *THOR* aligning with the MeRIP-seq results (GEO: GSE76367) and the MeRIP-qRT-PCR results showing that the m6A level on the lncRNA *THOR* was substantially decreased in the METTL3^stop/stop^ cell line, suggesting that METTL3 is the predominantly protein critical for m6A modification (Fig. 2). Additionally, the MeRIP-qRT-PCR assay revealed that four of six potential m6A sites are m6A modification sites (Fig. 4), revealing that m6A can maintain the oncogenic role of *THOR*.

m6A can alter the local structure of RNA and affect the binding of lncRNA to proteins^30,61^. The online prediction tool revealed different secondary structures for the four m6A mutations in the lncRNA *THOR* (Fig. 5), which is consistent with previous reports. Previous reports also indicated that the biological functions of m6A modifications are highly dependent on m6A “readers”, which selectively bind to methylated RNAs and determine the fates of transcripts^50^. In addition, cytoplasmic m6A modification readers include the YT521-B homology (YTH) domain family (YTHDF1-3)^1,62–64^. YTHDF1 regulates mRNA stabilization^31,65^, and YTHDF2 promotes mRNA decay in the cytoplasm^1,66^. YTHDF3 fine-tunes transcription by regulating RNA accessibility of YTHDF1 and YTHDF2^32,63,67^; therefore, we focused on YTHDF1 and YTHDF2 in this study^63,68^. Our RIP-qRT-PCR data and RNA interference assays confirmed that YTHDF1 and YTHDF2 can combine with lncRNA *THOR* in an m6A-dependent manner and regulate the fate of the lncRNAs (Fig. 6).

According to Hosono’s report, the lncRNA *THOR* can combine with IGF2BP1^10^ (a novel m6A reader^69^), while the results of the RNA pull-down/Western blot assay in our study showed that the 6A mutation cannot alter the combination of lncRNA *THOR* and IGF2BP1, and the results of the pull-down/MS assay demonstrated that the interaction between IGF2BP1 and lncRNA *THOR* did not depend on m6A modification (Fig. S5G, H). Furthermore, the qRT-PCR analysis revealed normal expression of the IGF2BP1 gene in the METTL3-, YTHDF1-, and YTHDF2-knockdown cells compared with the siNC control cells (Fig. S5I). In this study, we suggested that the motifs “GA (m6A) CA” (site 2 and site 3) can be read by YTHDF1^1,70^ and that the motifs “GG (m6A) CU” (site 4) and “UG (m6A) CU” (site 5) can be read by YTHDF2^2,70,71^. These results were consistent with previous studies showing that multiple mRNA transcripts containing the “GRAC” (R is G or A) motif can be read by YTHDF1, while the “G (m6A) CU” motif has the strongest affinity for YTHDF2 in the YTH family^1,2,72^.

In conclusion, we identified four m6A sites in the lncRNA *THOR* that interacted with m6A readers (including YTHDF1 and YTHDF2) to regulate the function of lncRNA *THOR* in a “*THOR*-m6A-reader” manner.

## Materials and methods

### Cell culture

Human 293T cells and A549 lung cancer cells were cultured in DMEM (Gibco Life Technologies, USA) containing 10% foetal bovine serum (FBS; Clark Bioscience, USA), and NCI-H1299 (H1299) cells were cultured in RPMI 1640 medium containing 10% foetal bovine serum. The stable h*THOR*^−/−^ and METTL3^stop/stop^ knockout H1299 cell lines were cultured in RPMI 1640 medium (Gibco Life Technologies, USA) containing 10% foetal bovine serum. The stable OE lacZ, OE WT, OE 6A-mutated, Maintain 1, Maintain 2, Maintain 3, Maintain 4, Maintain 5, Maintain 6, Mutation 2, Mutation 3, Mutation 4 and Mutation 5 overexpression H1299 cell lines were cultured in RPMI 1640 medium containing 10% foetal bovine serum.

### Cytoplasmic/nuclear RNA isolation and RNA-FISH

Nuclear and cytoplasmic RNA was isolated as previously described^15,73^. H1299 cells were collected and washed three times with cold PBS. The cells were subjected to lysis buffer containing 50 mM Tris-HCl, pH 8.0 (Meilunbio, China); 140 mM NaCl (Sigma-Aldrich, USA); 1.5 mM MgCl_2_ (Sigma-Aldrich, USA); 0.5% IGEPAL CA-630 (Sigma-Aldrich, USA); 1 U/μl RNase inhibitor (Thermo Fisher, USA); and 1 mM Dithiothreitol (DTT, Solarbio, China). The samples were mixed and incubated on ice for 5 min and centrifuged at 500 g for 3 min at 4 °C. The supernatant was poured into an RNase-free tube, and the precipitate was retained. The supernatant and precipitate were used for RNA extraction and qRT-PCR analysis. The primers used in this study are listed in Supplemental Table S1 (GENEWIZ, China).

The probes for RNA-FISH were purchased from GENEWIZ and were coupled spectrally to digoxin as reported previously with some minor modifications^10^. Cells were grown on 24-well chambered cover glasses and fixed with 4% paraformaldehyde solution (Boster, USA). The cells were incubated in a solution containing 50% formamide (Sigma-Aldrich, USA) and 5x SSC (Solarbio, China) for 5 min and then treated overnight with 10 nM FISH probes in 5x SSC containing 10% dextran sulfate (Sigma-Aldrich, USA), 0.02% RNase-free BSA (Solarbio, China), 0.25 mg/mL *E. coli* tRNA (Merck, USA) and 50% formamide at 37 °C. After hybridization, the cells were washed for 15 min with wash buffer (50% formamide in 5x SSC) at 55 °C and then washed again for 15 min with wash buffer (50% formamide in 2x SSC) at 55 °C. The cells were labelled with anti-digoxin antibodies (BIOSS, China) and incubated overnight at 4 °C. The following day, the cells were stained for 60 min with Fluor-594-labelled goat anti-rabbit IgG (BIOSS, China) at room temperature. The nuclei were stained with DAPI (Thermo Scientific, USA) for 5 min at room temperature. The images were captured using a fluorescence microscope (Zeiss LSM800 confocal microscope). The probes and antibodies used in this study are listed in Supplemental Tables S2 and S3, respectively.

### Plasmid construction

To construct the *THOR*-knockout cell line, sgRNAs were inserted into a px458 vector (http://www.addgene.org/48138/), as previous study^74^. The METTL3 stop codon sgRNA was inserted into a 74707 vector as previous study^75^. The sgRNAs used in this study can be found in Supplemental Table S2 (GENEWIZ, China).

pcDNA3.1_*THOR* and pcDNA3.1_mut*THOR* (6A mutation) vectors were purchased from GenScript (Nanjing, China). Wild-type *THOR* exons and 6A-mutated *THOR* exons in the two vectors were amplified. The OE 6A-mutated *THOR* exons were used to obtain single-site maintenance (Maintain 1, Maintain 2, Maintain 3, Maintain 4, Maintain 5, and Maintain 6) cells via a Mut Express II Fast Mutagenesis Kit V2 (Vazyme, Nanjing, China), and the OE WT *THOR* exons were used to obtain single-mutation *THOR* exons (Mutation 2, Mutation 3, Mutation 4 and Mutation 5). All *THOR* exons were inserted into a PiggyBac Dual promoter (PB513B-1 vector, Miaolingbio, China) to generate stable overexpressing cell lines. A lacZ-overexpressing plasmid, in which each WT *THOR* exon was replaced with a lacZ sequence, was used as a control.

### RNA interference and plasmid transfection

For RNA interference, the cells were transfected with 30 nM siRNA (GenePharma, China) targeting the lncRNA *THOR*, METTL3, YTHDF1, or YTHDF2 and a control siRNA using Lipofectamine RNAiMAX (Thermo Fisher, 3778150, USA) following the manufacturer’s instructions. DNA was isolated and performed to PCR and Sanger Sequencing analysis after 48 h later. The siRNA sequences are listed in Supplemental Table S2.

For DNA plasmid transfection, Lipofectamine^TM^ 3000 (Thermo Fisher, L3000015, USA) was used as suggested by the manufacturer.

### Genotyping, RNA extraction and real-time quantitative PCR

Genomic DNA was isolated using a TIANamp genomic DNA kit (TIANGEN, Beijing, China). The DNA was amplified via PCR with 2× PrimeSTAR Max Premix (TaKaRa Bio, Tokyo, Japan), and the PCR primers used to detect these mutations are shown in Supplemental Table S1.

For qRT-PCR analyses, total RNA was extracted from cells with TRIzol reagent (Invitrogen, USA), and qRT-PCR was performed according to the protocol of a previous study^76^. cDNA was synthesized using a FastKing RT kit (with gDNase) (Tiangen Biotech, China) according to the manufacturer’s recommendations. qPCR assays were performed using SuperReal PreMix Plus (SYBR Green) (TIANGEN Biotech, China) according to the manufacturer’s instructions and a Bio-Rad Iq5 Multicolor Real-Time PCR detection system. The expression levels of the treated samples were normalized to the level of the controls, with *GAPDH* or *U6* serving as the endogenous control, and were calculated by the 2^−ΔΔCT^ formula. The primers used in this study are presented in Supplemental Table S1 (GENEWIZ, China).

### Protein isolation and western blotting

Total protein was extracted from cells using radioimmunoprecipitation assay lysis buffer supplemented with phenylmethanesulfonyl fluoride (PMSF, Roche Applied Science, USA) and phosphatase inhibitor (PI, Thermo Scientific, USA). The protein concentration was measured using an enhanced BCA protein assay kit (Beyotime, P0010, China). Total protein extracts were separated on 10% or 12% gels via SDS-PAGE and then transferred to 0.22 nm polyvinylidene fluoride membranes (Millipore, USA). The proteins were probed with specific antibodies after the blot was blocked with 5% non-fat milk (Boster, AR0104, USA). The antibodies used in this study are listed in Supplemental Table S3.

### Plate formation assay and soft-agar colony formation assay

Five hundred cells were mixed in 2 ml of culture medium and plated onto 6-well plates. The plates were incubated at 37 °C with 5% CO_2_, and the medium was replaced for each week. After 3–4 weeks, the colonies were fixed in 4% paraformaldehyde solution, then stained using 0.1% crystal and counted.

A 1.5 ml layer of 0.7% NuSieve GTG agarose (Lonza, 50081) was plated into the bottom of 6-well plates. Then, 1.5 ml of cell mixture containing 10^4^ cells in culture medium and a final concentration of 0.35% agarose was carefully plated on top of the bottom laye, and incubated at 37 °C and 5% CO_2_. After 4 weeks, colonies were treated with 4% paraformaldehyde solution, stained using 0.1% crystal (Meilunbio, China) and counted.

### Cell migration and invasion assay in vitro

The cell migration assays were performed as follows. A total of 10^5^ cells suspended in serum-free medium were placed into the upper chamber (Corning, 3422, USA), and culture medium with 10% foetal bovine serum was added to the lower chamber. The cells remaining on the upper surface of the membrane were removed after 6 h.

For the invasion assays, the experimental procedures were similar to the migration assays, except that the filters were coated with Matrigel (BD Biosciences, USA), and 10% foetal bovine serum culture medium were used. The Transwell plates were incubated at 37 °C and 5% CO_2_ for 24 h. The cells in the top chambers were removed with cotton swabs.

The migrated and invaded cells on the lower membrane surface were washed twice with PBS buffer and fixed in 4% paraformaldehyde solution for 30 min at room temperature. The well was stained with 0.1% crystal violet for 30 min using PBS buffer, and more than three times wash in ddH_2_O, the membrane was dried and observed under a microscope. The assays were performed in triplicate.

### Wound-healing assay

The wound-healing assays were performed as follows. The H1299 cells were seeded in dishes with a wound-healing plugin and cultured to 100% confluence. A wound was then produced in the cell monolayer by moving a wound-healing insert (ibidi, Germany). The cells were then washed in PBS buffer and cultured for another 24 h, and the cells were observed and imaged under a microscope.

### Xenograft tumours

Nude mice were handled and maintained according to the National Institutes of Health Animal Care and the Use Committee guidelines of the Animal Care Center and Use Committee of Jilin University (Changchun, China). Female nude mice (4 weeks old, 18–20 g) were purchased from Liaoning Changsheng Biotechnology (Liaoning, China), and all tumour-bearing nude mice were randomly allocated to groups with 5 nude mice per group. In general, 2 × 10^6^ cells were suspended in 200 μl of serum-free RPMI 1640 for injection into each mouse. The right groin of each nude mouse was then subcutaneously injected with cells to establish a xenograft. After the tumours formed, the minimum width (W) and maximum length (L) of each tumour was measured weekly with callipers. The animals were sacrificed 4–5 weeks after the injection.

### Methylated RNA immunoprecipitation-qRT-PCR assay

To determine the m6A modifications of individual genes, we performed the Methylated RNA immunoprecipitation (MeRIP) assay with a Magna MeRIP™ m6A kit (Sigma-Aldrich, USA) according to the manufacturer’s instructions. The collected solutions were used to confirm the m6A sites via qRT-PCR assay. The primers used in this study are presented in Supplemental Table S1.

### RNA decay assays

To measure RNA stability, the half-life of *THOR* was measured as previously described^61^. In brief, 5 μg/ml actinomycin D (Act-D, Sigma-Aldrich, USA) was added to the cells. After the cells were harvested, and RNA was isolated with TRIzol for use in qRT-PCR. The half-life of the lncRNA *THOR* was calculated with *GAPDH* used for normalizing the data. The primers used in this study are presented in Supplemental Table S1.

### RNA pull-down/western blot/mass spectrometry analyses

WT and 6A-mutated lncRNA *THOR* sequences were obtained via PCR and used as templates for the RNA pull-down assay. RNA pull-down assays were performed using the Pierce™ RNA 3′ end Desthiobiotinylation Kit (Thermo Scientific, USA) and Pierce™ magnetic RNA-protein pull-down kit (Thermo Scientific, USA) according to the manufacturer’s instructions. Western blotting was performed as previously described^77^, the antibodies used in this study are listed in Supplemental Table S3. Finally, the mass spectrometry (MS) analysis was performed by the GeneCreate Biological Engineering Co., Ltd. (Wuhan, China). The results of the MS are presented in Supplemental Table S4.

### RNA immunoprecipitation-qRT-PCR assay

The RNA immunoprecipitation (RIP) assay was performed as described previously^15,73^. OE WT cells were collected and subsequently lysed with RIP lysis buffer. The beads (Thermo Scientific, USA) were combined with YTHDF1 (Proteintech, 17479-1-AP, China) or YTHDF2 (Proteintech, 24744-1-AP, China) antibody and washed twice with RIP wash buffer. Lysis buffer and beads were incubated overnight with the lysed cells at 4 °C. The following day, the beads were collected and washed with RIP wash buffer, and the RNA complexes were isolated through phenol-chloroform extraction and analysed via qRT-PCR. The primers used in this study are listed in Supplemental Table S1.

### Statistics analysis

By using Prism 8.0 (GraphPad), the significant differences were determined via a two-tailed unpaired Student’s *t*-test or analysis of variance. The data are expressed as the mean ± s.e.m. *, #*p* < 0.05, **, ##*p* < 0.01, ***, ###*p* < 0.001 and ****p < 0.0001 denote the significance thresholds; ns denotes not significant.

## Supplementary information

Figure S1

Figure S2

Figure S3

Figure S4

Figure S5

Supplemental Figure legends

Supplemental Table S1

Supplemental Table S2

Supplemental Table S3

Supplemental Table S4
