# Supplementary figures and images for: A novel N6-methyladenosine (m6A)-dependent fate decision for the lncRNA THOR
Source: Cell Death Dis. 2020 Aug 13;11(8):613. doi: 10.1038/s41419-020-02833-y (PMC7426843; doi:10.1038/s41419-020-02833-y)

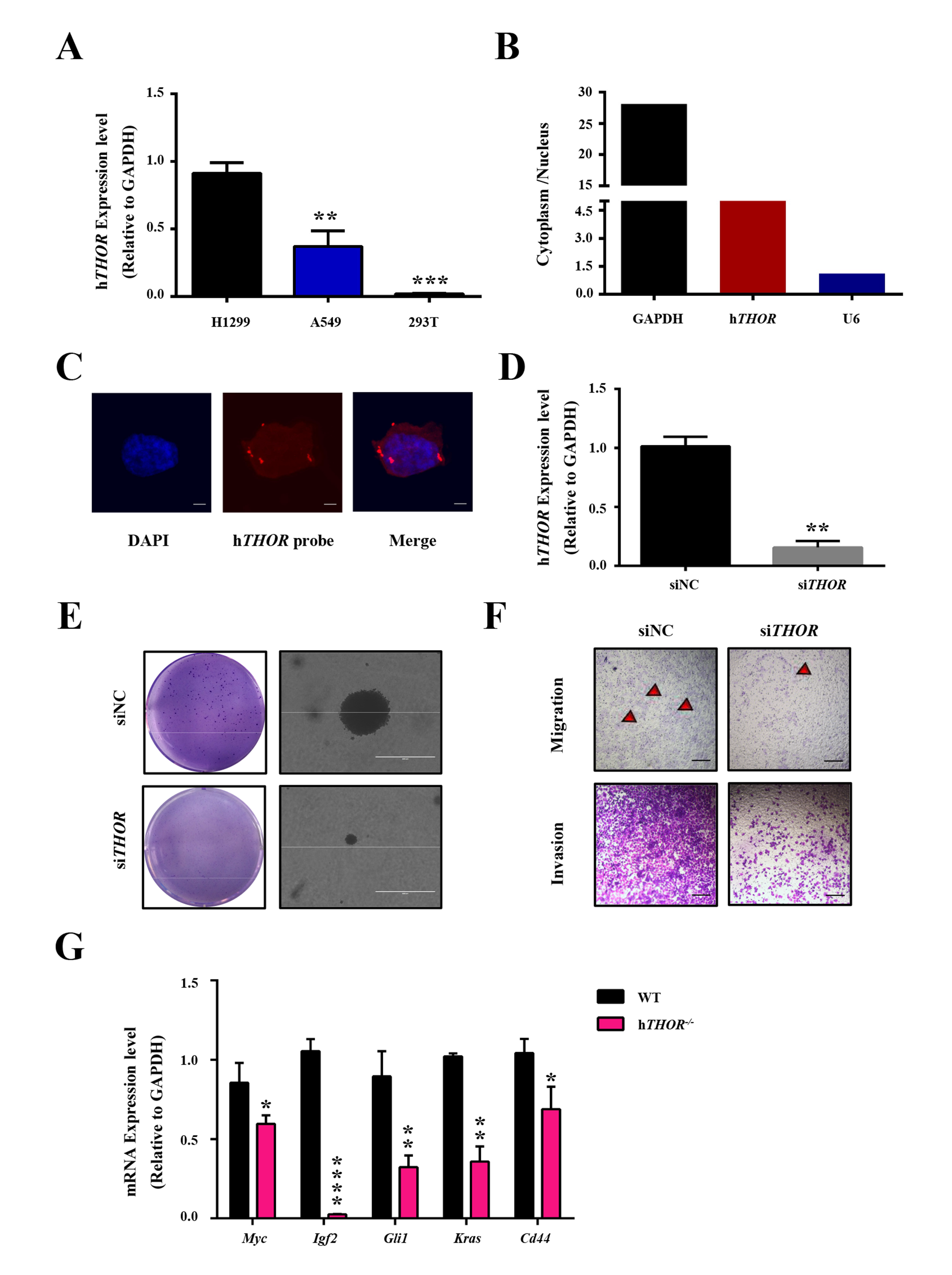

Supplement: Supplementary file 1 — Figure S1 [file 41419_2020_2833_MOESM1_ESM.tif]

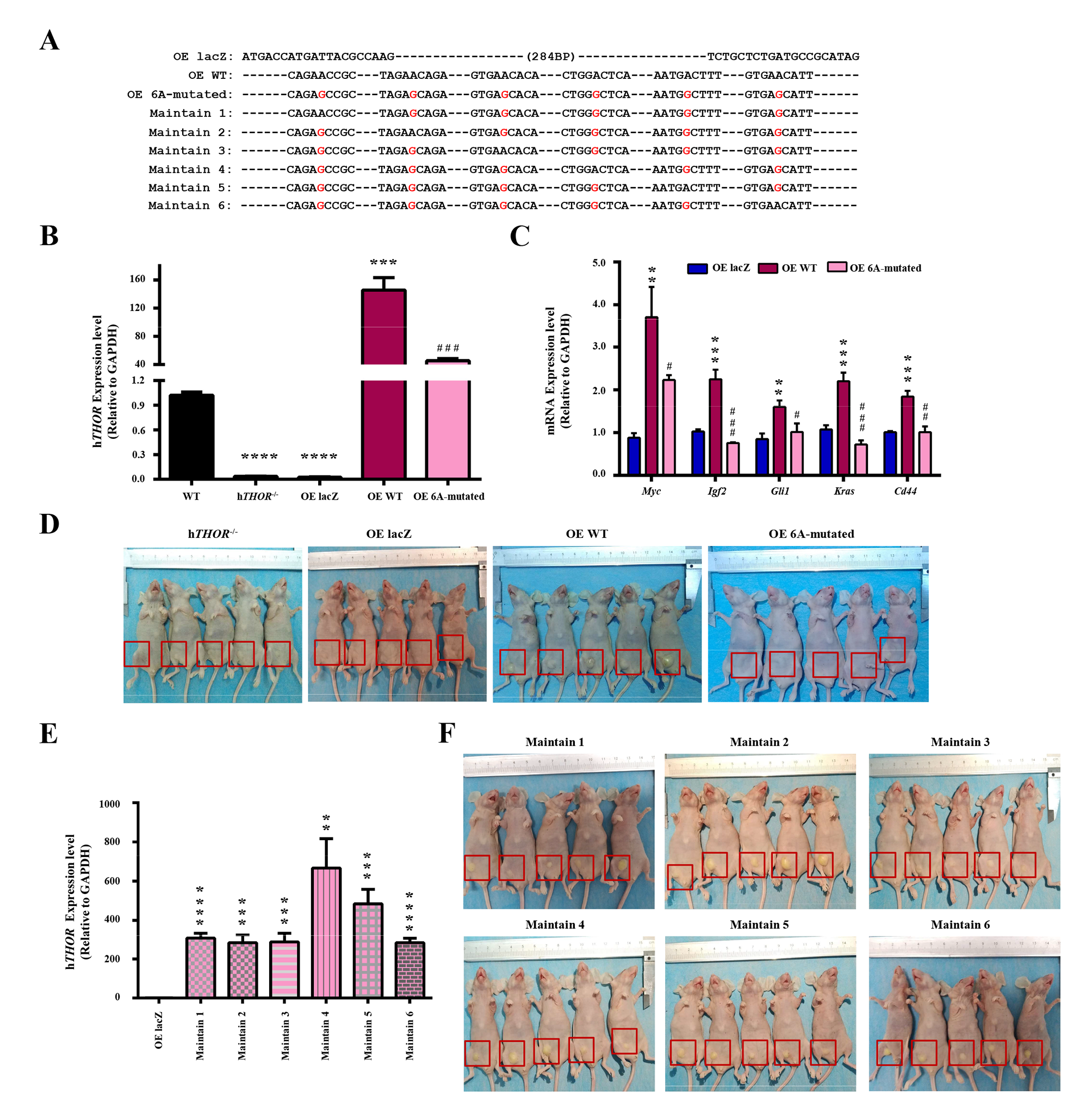

Supplement: Supplementary file 2 — Figure S2 [file 41419_2020_2833_MOESM2_ESM.tif]

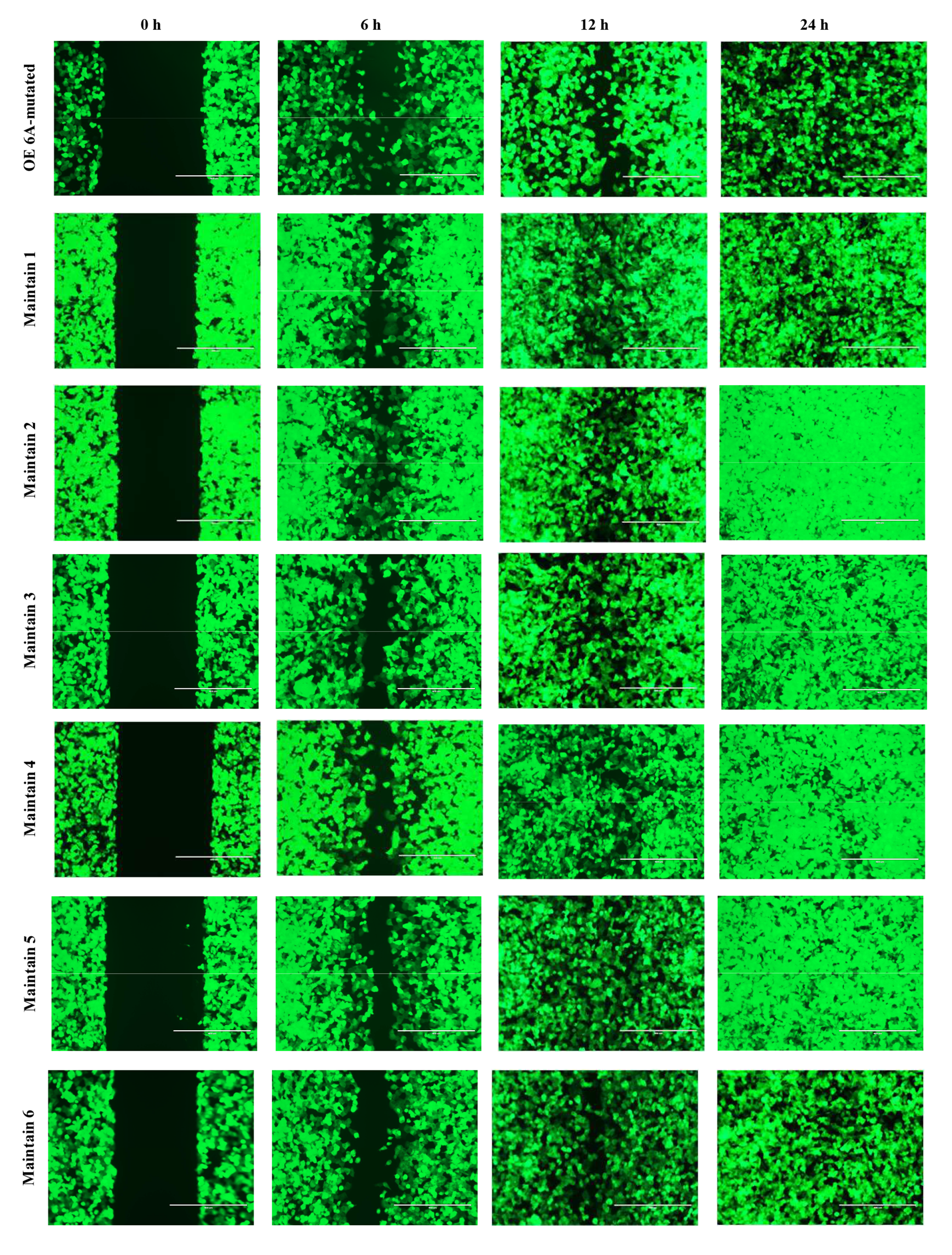

Supplement: Supplementary file 3 — Figure S3 [file 41419_2020_2833_MOESM3_ESM.tif]

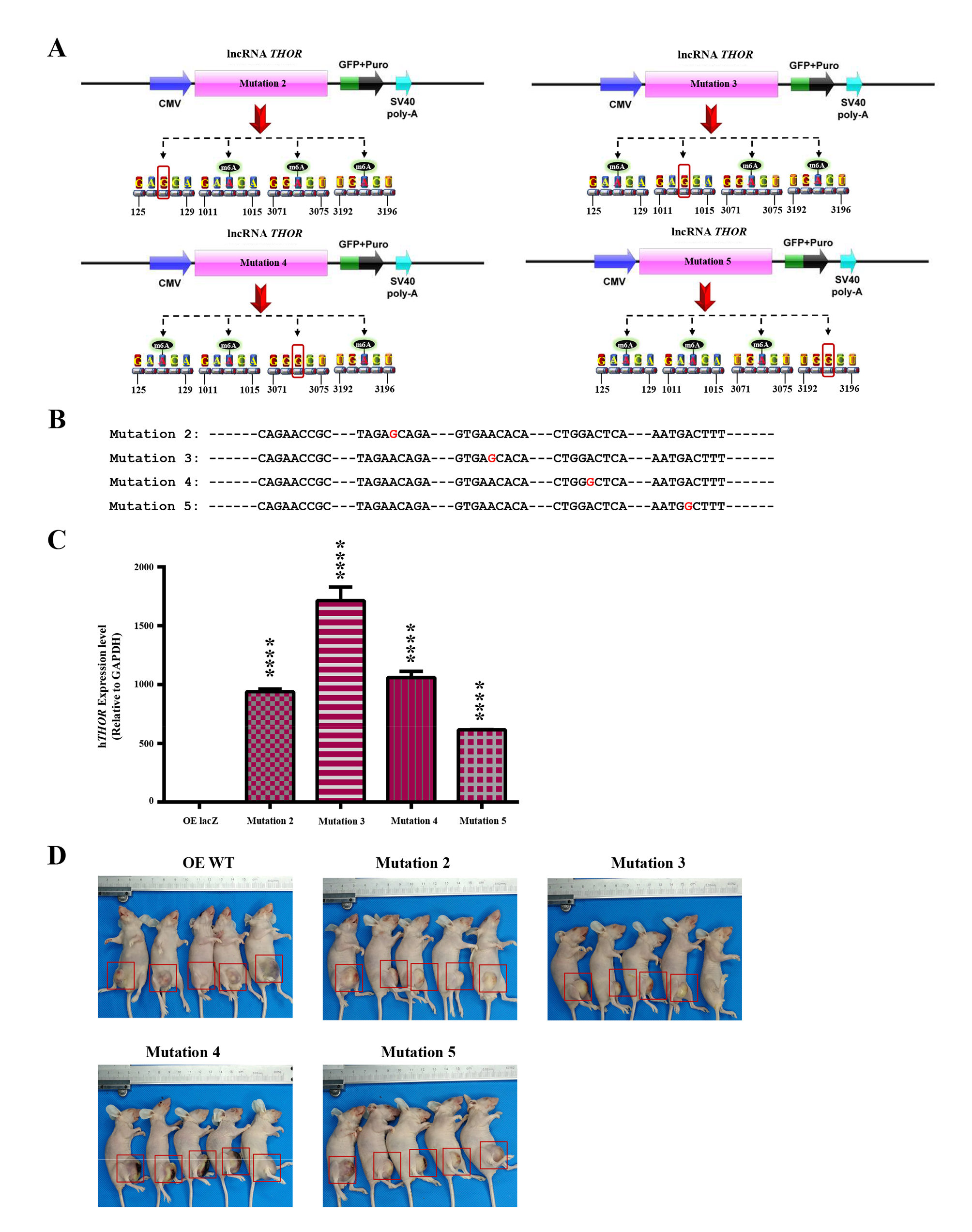

Supplement: Supplementary file 4 — Figure S4 [file 41419_2020_2833_MOESM4_ESM.tif]

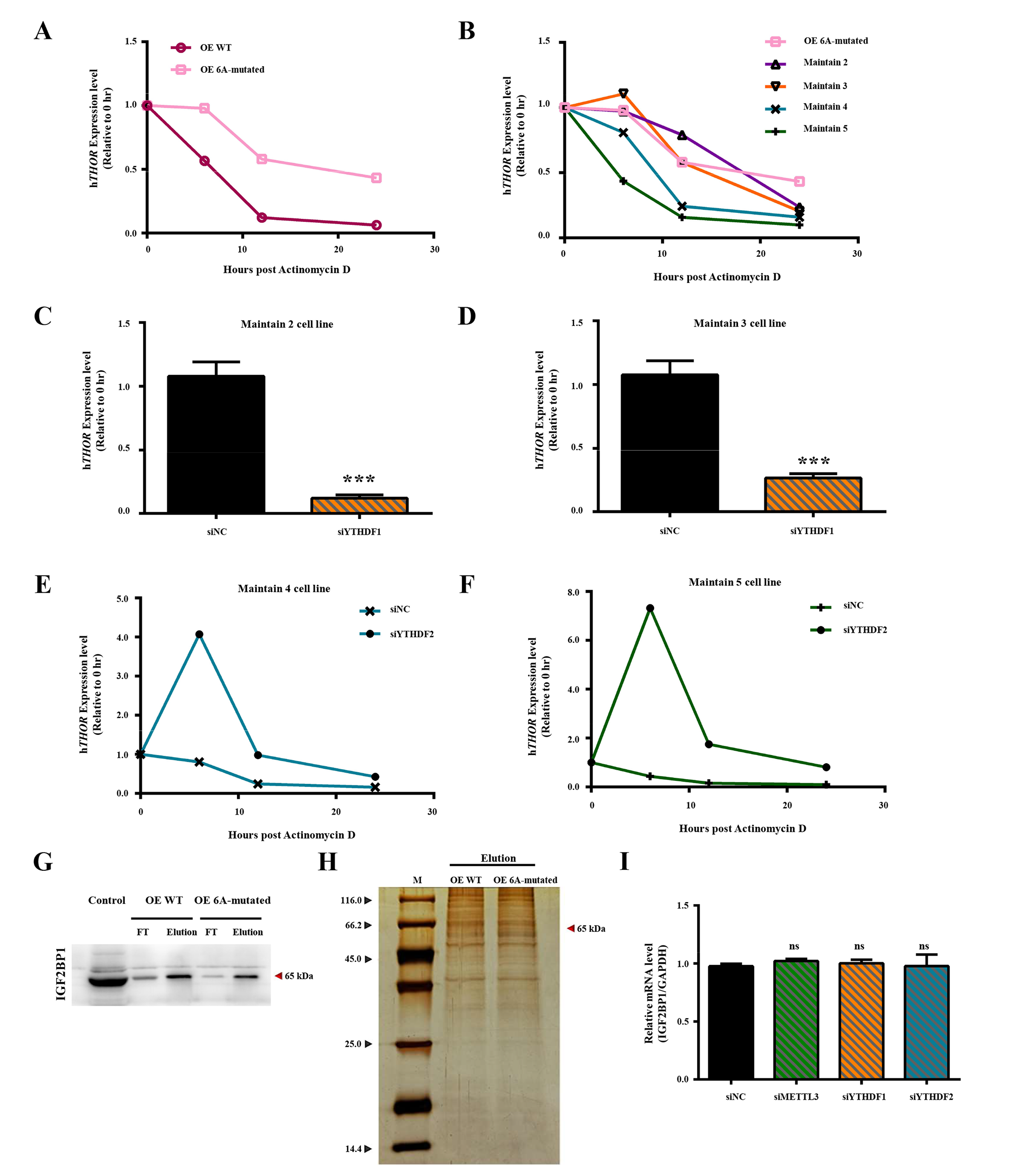

Supplement: Supplementary file 5 — Figure S5 [file 41419_2020_2833_MOESM5_ESM.tif]
