# Supplementary material for: A novel N6-methyladenosine (m6A)-dependent fate decision for the lncRNA THOR
Source: Cell Death Dis. 2020 Aug 13;11(8):613. doi: 10.1038/s41419-020-02833-y (PMC7426843; doi:10.1038/s41419-020-02833-y)
Supplement: Supplementary file 6 — Supplemental Figure legends [file 41419_2020_2833_MOESM6_ESM.docx]

**Fig. S1. Significantly reduced cell proliferation in h*THOR*^-/-^ cells.**

A. The expression of lncRNA *THOR* in H1299, A549 and 293T cells was determined by qRT-PCR assays (^**^*p*<0.01, ^***^*p*<0.001).

B. The expression of lncRNA *THOR* transcripts in cytoplasmic or nuclear fractions of human cells was determined by qRT-PCR. The results showed that lncRNA *THOR* was expressed in the nucleus and was enriched in the cytoplasm.

C. Cytoplasmic and nuclear expression of lncRNA *THOR* in H1299 cells was assessed by RNA-FISH. The results showed that lncRNA *THOR* exists in the nucleus and cytoplasm. Scar bars, 50 μm.

D. The significantly decreased expression of lncRNA *THOR* in si*THOR* H1299 cells by qRT-PCR (^**^*p*<0.01).

E. Plate colony formation assays and soft agar assays of si*THOR*-treated and control cells; the results indicate reduced cell proliferation in lncRNA *THOR* knockdown cells. Scar bars, 400 μm.

F. The reduced migration and invasion ability of si*THOR*-treated cells compared with control cells. The migration and invasion of si*THOR*-treated and control cells was analysed using polycarbonate membrane inserts in a 24-well plate. Scar bars, 50 μm.

G. The expression of *Myc*, *Igf2*, *Gli1*, *Kras*, and *Cd44* in h*THOR*^-/-^ and WT cells were determined by qRT-PCR assays (^*^*p*<0.05, ^**^*p*<0.01, and ^****^*p*<0.0001).

**Fig. S2. The determination of potential m6A modification function and accurate sites in lncRNA *THOR*.**

A. The Sanger sequencing of OE lacZ, OE WT, OE 6A-mutated and Maintain 1-6 cell lines. The point mutation is shown in red.

B. Downregulated expression of lncRNA *THOR* in h*THOR*^-/-^ and OE lacZ cell lines compared with that of WT cells, which was determined by qRT-PCR assays (^****^*p*<0.0001); Upregulated expression of lncRNA *THOR* in OE WT, OE 6A-mutated cells compared with that of WT cells, which was determined by qRT-PCR assays (^***^*p*<0.001); downregulated expression of lncRNA *THOR* in OE 6A-mutated cells compared with that of OE WT cells, which was determined by qRT-PCR assays (^###^*p*<0.001).

C. The expression of *Myc*, *Igf2*, *Gli1*, *Kras*, and *CD44* in OE lacZ, OE WT, and OE 6A-mutated cell lines was determined by qRT-PCR assays. The asterisk (*) denotes a significant difference from OE lacZ, and the hash mark (#) denotes a significant difference from OE WT (^*^*p*<0.05, ^**, ##^*p*<0.01, and ^***, ###^*p*<0.001).

D. Photos of tumour xenograft assay for h*THOR*^-/-^, OE lacZ, OE WT, and OE 6A-mutated cell lines. The subcutaneous tumours are indicated by a red box.

E. Upregulated expression of lncRNA THOR in Maintain 1-6 cell lines compared with that of OE lacZ cells, which was determined by qRT-PCR assays (**p<0.01, ^***^p<0.001, and ^****^p<0.0001).

F. Photos of tumour xenograft assay for Maintain 1-6 cell lines. The subcutaneous tumours are indicated by a red box.

**Fig. S3. The wound healing assays to determine the accurate m6A modification sites in lncRNA *THOR*.**

Wound healing assay results for OE 6A-mutated cells and Maintain 1-6 cells. The scratch was measured 6, 12, and 24 hours after it was initially made. Scar bars, 400 μm.

**Fig. S4. Schematic of m6A site mutation cell lines and tumour xenograft assay.**

A. Schematic diagram of overexpression (OE) plasmids with single m6A site mutation (Mutation 2-5).

B. Sanger sequencing results of Mutation 2, 3, 4, and 5 cell lines. The point mutation is shown in red.

C. Upregulated expression of lncRNA *THOR* in Mutation 2-5 cell lines compared with that of OE lacZ cells, which was determined by qRT-PCR assays (^****^*p*<0.0001).

D. Photos of tumour xenograft assay results from Mutation 2, 3, 4, and 5 cell lines. The subcutaneous tumours are shown by a red box.

**Fig. S5. The m6A readers YTHDF1 and YTHDF2 play balancing roles in regulating the gene transcription or decay of the lncRNA *THOR*.**

A. The expression level of lncRNA *THOR* following Actinomycin D treatment in OE WT and OE 6A-mutated cells was detected by qRT-PCR assay, showing m6A modification can reduce the half-life of lncRNA *THOR*.

B. The expression level of lncRNA *THOR* following Actinomycin D treatment in Maintain 2, Maintain 3, Maintain 4, and Maintain 5 cells was detected by qRT-PCR assay, showing fourth and fifth m6A modification can reduce the half-life of lncRNA *THOR*.

C. The expression level of lncRNA *THOR* following siYTHDF1 in Maintain 2 cells was detected by qRT-PCR assay, showing the expression of lncRNA *THOR* was reduced in Maintain 2 cell line when compared with siNC cells (^***^*p*<0.001).

D. The expression level of lncRNA *THOR* following YTHDF1 interference in Maintain 3 cells was detected by qRT-PCR assay, showing the expression of lncRNA *THOR* was reduced in Maintain 3 cell line when compared with siNC cells (^***^*p*<0.001).

E. The expression level of lncRNA *THOR* following Actinomycin D treatment in siNC- and siYTHDF2-treated Maintain 4 cells was detected by qRT-PCR assay, showing YTHDF2 interference can prolong the half-life of lncRNA *THOR* and YTHDF2 can read the fourth m6A site.

F. The expression levels of lncRNA *THOR* following Actinomycin D treatment in siNC- and siYTHDF2-treated Maintain 5 cells was detected by qRT-PCR assay, showing YTHDF2 interference can prolong the half-life of lncRNA *THOR* and YTHDF2 can read the fifth m6A site.

G. RNA pull-down analysis and western blot identified IGF2BP1 as a specific protein interacting with lncRNA *THOR* not dependent on m6A modification.

H. RNA pull-down analysis and MS identified m6A modification can’t affected the interaction between IGF2BP1 and lncRNA *THOR*. The red arrow shows the position of IGF2BP1.

I. The expression of IGF2BP1 in siMETTL3, siYTHDF1, siYTHDF2 cells and siNC cells was determined by qRT-PCR. The results showed that the expression of IGF2BP1 was not changed in all cells compared with that of control cells (siNC) (ns denotes not significant).
