# Supplementary material for: A novel N6-methyladenosine (m6A)-dependent fate decision for the lncRNA THOR
Source: Cell Death Dis. 2020 Aug 13;11(8):613. doi: 10.1038/s41419-020-02833-y (PMC7426843; doi:10.1038/s41419-020-02833-y)
Supplement: Supplementary file 7 — Supplemental Table S1 [file 41419_2020_2833_MOESM7_ESM.docx]

|  | **Application** | **Forward (5'-3')** | **Reverse (5'-3')** |
| --- | --- | --- | --- |
| h*THOR* | qRT-PCR | CAAGGTGCTTCTCTCTGGATTT | GCCAAAGTCATTTGTTGGGTAT |
| hGAPDH | qRT-PCR | GGTGAAGGTCGGAGTCAACG | TGGGTGGAATCATATTGGAACA |
| hU6 | qRT-PCR | CTCGCTTCGGCAGCACA | AACGCTTCACGAATTTGCGT |
| YTHDF1 | qRT-PCR | AGCACACAACCTCCATCTTC | TGTTTCGACTCTGCCGTTC |
| YTHDF2 | qRT-PCR | CCAGTGGGATTGACTTCTCAG | GGAGCTAGGTGCATAAGCATAA |
| MYC | qRT-PCR | CTTCTCTCCGTCCTCGGATTCT | GAAGGTGATCCAGACTCTGACCTT |
| IGF2 | qRT-PCR | GCGGCTTCTACTTCAGCAG | CAGGTGTCATATTGGAAGAAC |
| GLI1 | qRT-PCR | AGGGAGTGCAGCCAATACAG | ATTGGCCGGAGTTGATGTAG |
| KRAS | qRT-PCR | ACACAAAACAGGCTCAGGACT | AGGCATCATCAACACCCTGT |
| CD44 | qRT-PCR | AGAAGGTGTGGGCAGAAGAA | AAATGCACCATTTCCTGAGA |

**Table S1. The primers of PCR and qRT-PCR used in this study.**

|  | **Application** | **Forward (5'-3')** | **Reverse (5'-3')** |
| --- | --- | --- | --- |
| Site 1 | MeRIP-qRT-PCR | GGTAGGTGCTGCCATGC | CCGGAGAGATGGCTTTGTTT |
| Site 2 | MeRIP-qRT-PCR | AGAACCGCACGAAAGCAAA | ACCAGGACGGCAGGAAATA |
| Site 3 | MeRIP-qRT-PCR | CCGGAGCAGAAATAGAACAGAC | TCTGAGTCCAGCCACCTAAT |
| Site 4 | MeRIP-qRT-PCR | AATCGAGCAAGGCAGTGAA | AATCCAGAGAGAAGCACCTTG |
| Site 5 | MeRIP-qRT-PCR | TTCACCTGCCTTGCCAAA | GGCCAAGACCTGCTGTTAG |
| Site 6 | MeRIP-qRT-PCR | GAAGAGTTAAGGCACCATCTGT | CCAGACATCCATCACTGCTAAA |
| h*THOR* | MeRIP-qRT-PCR | CAAGGTGCTTCTCTCTGGATTT | GCCAAAGTCATTTGTTGGGTAT |
| hGAPDH | MeRIP-qRT-PCR | GGTGAAGGTCGGAGTCAACG | TGGGTGGAATCATATTGGAACA |

|  | **Application** | **Forward (5'-3')** | **Reverse (5'-3')** |
| --- | --- | --- | --- |
| h*THOR* (primer1) | RIP-qRT-PCR | CAAGGTGCTTCTCTCTGGATTT | GCCAAAGTCATTTGTTGGGTAT |
| h*THOR* (primer2) | RIP-qRT-PCR | GGCAGTGAAGCAAACATCATTAG | TTGGCAAGGCAGGTGAAA |

|  | **Application** | **Forward (5'-3')** | **Reverse (5'-3')** |
| --- | --- | --- | --- |
| h*THOR*^-/-^ (genotyping) | PCR | TGGAAGAGCCATTCCCATTT | GGACACTTTCAAGGAAGCATTT |
| METTL3^stop/stop^(genotyping) | PCR | TGTCCGCGTGAGAATTGG | TGAGCAGCCTCACAAAGG |
| OE lacZ, OE WT, and OE 6A-mutated (genotyping) | PCR | ACATCAATGGGCGTGGATAG | GCCAGTACACGACATCACTT |
| Maintain 1-6, Mutation 2,3,4 and 5(genotyping) | PCR | ACATCAATGGGCGTGGATAG | GCCAGTACACGACATCACTT |
