# Supplementary material for: A novel N6-methyladenosine (m6A)-dependent fate decision for the lncRNA THOR
Source: Cell Death Dis. 2020 Aug 13;11(8):613. doi: 10.1038/s41419-020-02833-y (PMC7426843; doi:10.1038/s41419-020-02833-y)
Supplement: Supplementary file 8 — Supplemental Table S2 [file 41419_2020_2833_MOESM8_ESM.docx]

**Table S2. The sequences of probes, sgRNAs and siRNAs used in this study.**

|  | **Application** | **5'-3'** |
| --- | --- | --- |
| THOR-probe-1 | RNA FISH | TTGCTTTCGTGCGGTTCTGCGA |
| THOR-probe-2 | RNA FISH | TCACTGCCTTGCTCGATTGT |
| THOR-probe-3 | RNA FISH | GTTGGGTATTGCCGGACAC |

|  | **Application** | **Forward (5'-3')** | **Reverse (5'-3')** |
| --- | --- | --- | --- |
| hTHOR-sgRNA-1 | CRISPR | CACCgAGGGTGTAGCGCGGGCTAGA | AAACTCTAGCCCGCGCTACACCCTc |
| hTHOR-sgRNA-2 | CRISPR | CACCgCATGCAGTGAATAGTTTTAG | AAACCTAAAACTATTCACTGCATGc |
| METTL3-stop-sgRNA | CRISPR | CACCgGAAGCAGCTGGACTCTCTGC | AAACGCAGAGAGTCCAGCTGCTTCc |

|  | **Application** | **Forward (5'-3')** | **Reverse (5'-3')** |
| --- | --- | --- | --- |
| siNC | RNA interference | UUCUCCGAACGUGUCACGUTT | ACGUGACACGUUCGGAGAATT |
| siTHOR | RNA interference | CUAUGGUGUGUGAACAUUATT | UAAUGUUCACACACCAUAGTT |
| siYTHDF1-370 | RNA interference | GGAUACAGUUCAUGACAAUTT | AUUGUCAUGAACUGUAUCCTT |
| siYTHDF1-1503 | RNA interference | GCUCCAUUAAGUACUCCAUTT | AUGGAGUACUUAAUGGAGCTT |
| siYTHDF2-624 | RNA interference | GCCCAAUAAUGCAUAUACUTT | AGUAUAUGCAUUAUUGGGCTT |
| siYTHDF2-1133 | RNA interference | GCGGGUCCAUUACUAGUAATT | UUACUAGUAAUGGACCCGCTT |
| SiMETTL3 | RNA interference | GCAAGUAUGUUCACUAUGATT | UCAUAGUGAACAUACUUGCAG |
