# Supplementary material for: A novel N6-methyladenosine (m6A)-dependent fate decision for the lncRNA THOR
Source: Cell Death Dis. 2020 Aug 13;11(8):613. doi: 10.1038/s41419-020-02833-y (PMC7426843; doi:10.1038/s41419-020-02833-y)
Supplement: Supplementary file 9 — Supplemental Table S3 [file 41419_2020_2833_MOESM9_ESM.docx]

**Table S3. The antibodies used in this study.**

| **Antibody** | **Company** | **Cat. Number** | **source** |
| --- | --- | --- | --- |
| Anti-Digoxin/AF594 | BIOSS | bs-0356R-AF594 | Rabbit |
| METTL3 | Proteintech | 15073-1-AP | Rabbit |
| β-Tublin | Proteintech | 66240-1-lg | Mouse |
| YTHDF1 | Proteintech | 17479-1-AP | Rabbit |
| YTHDF2 | Proteintech | 24744-1-AP | Rabbit |
| IGF2BP1 | Proteintech | 22803-1-AP | Rabbit |
| CoraLite594 – conjugated Goat Anti-Rabbit IgG(H+L） | Proteintech | SA-00013-4 | Aniti-Rabbit |
| HRP-conjugated Affinipure Goat Anti-Mouse IgG(H+L） | Proteintech | SA00001-1 | Aniti-Mouse |
| HRP-conjugated Affinipure Goat Anti-Rabbit IgG(H+L） | Proteintech | SA00001-2 | Aniti-Rabbit |
